# Supplementary material for: PARP Theranostic Auger Emitters Are Cytotoxic in BRCA Mutant Ovarian Cancer and Viable Tumors from Ovarian Cancer Patients Enable Ex-Vivo Screening of Tumor Response
Source: Molecules. 2020 Dec 19;25(24):6029. doi: 10.3390/molecules25246029 (PMC7766161; doi:10.3390/molecules25246029)
Supplement: Supplementary file 1 [file molecules-25-06029-s001.pdf]

Supplementary Figures

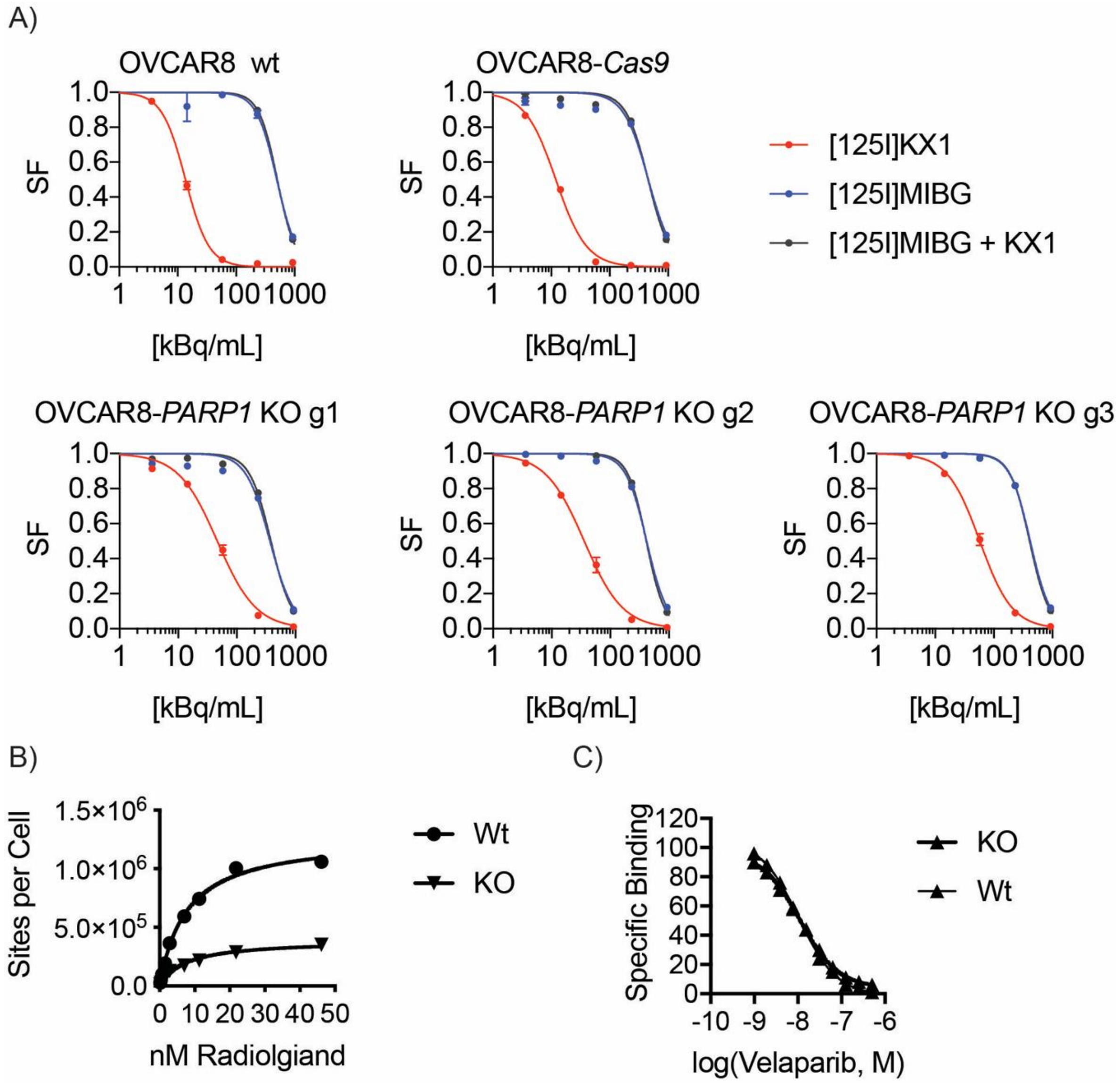

Sup Figure 1. Binding specificity and toxicity of [^{125}I]KX1. **A)** Cell survival curves showing response of OVCAR8 and OVCAR8 *PARP1* KO cells to [^{125}I]KX1, [^{125}I]MIBG, and [^{125}I]MIBG + nonradiolabeled KX1. **B)** Saturation radiobinding assay of [^{125}I]KX1 binding to OVCAR8 WT and *PARP1* KO cells. (C) Competition radioligand assay of veliparib.

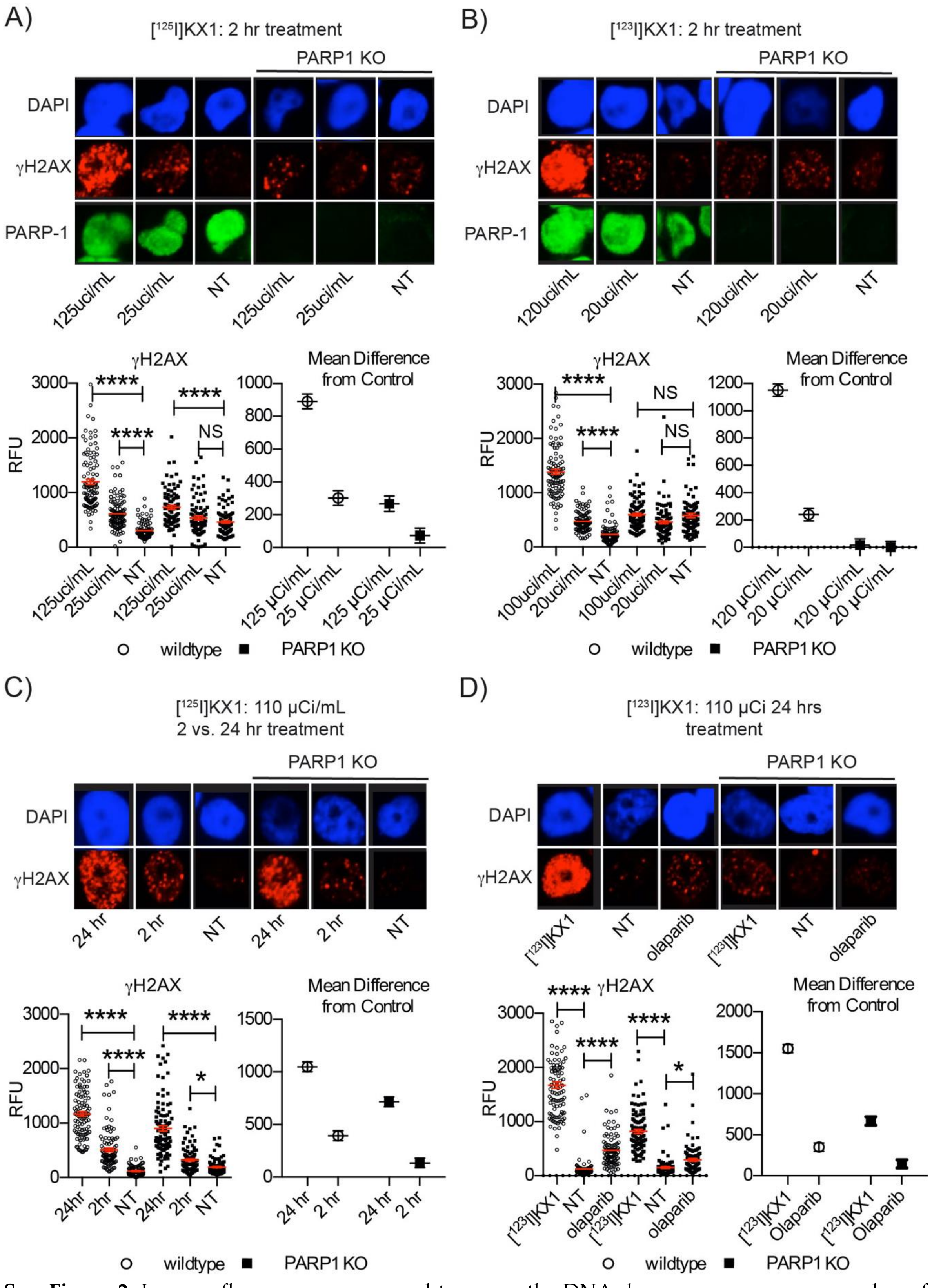

Sup Figure 2. Immunofluorescence was used to assess the DNA damage response as a marker of drug target specificity in OVCAR8 wildtype (wt) and *PARP1* KO cells treated with either [^{125}I]KX1 or [^{123}I]KX1. **A)** OVCAR8 wt cells treated for 2 h with [^{125}I]KX1 showed a dose dependent increase in DNA damage marker γH2AX, while OVCAR8 *PARP1* KO cells only showed a significant increase at the highest dose tested. **B)** Similarly, [^{123}I]KX1 showed a dose dependent induction of γH2AX in OVCAR8 wt treated cells but not OVCAR8 *PARP1* KO cells at either concentrations evaluated. **C)** DNA damage induced by [^{125}I]KX1 in OVCAR8 cells showed a time dependent increase although the magnitude of the increase was greater for wt compared to *PARP1* KO cells. **D)** OVCAR8 cells treated

with either [^{123}I]KX1 or non-radioactive PARP inhibitor olaparib also showed a greater response in wt cells compared to *PARP1* KO. Collectively this data demonstrated that [$^{123/125}$I]KX1 induced DNA damage that is specific to the drug target PARP-1. All experimental data was statistically evaluated using ANOVA analysis and statistical significance has been denoted with corresponding p-values * <0.05, ** <0.01, ***<0.001, ****<0.0001.

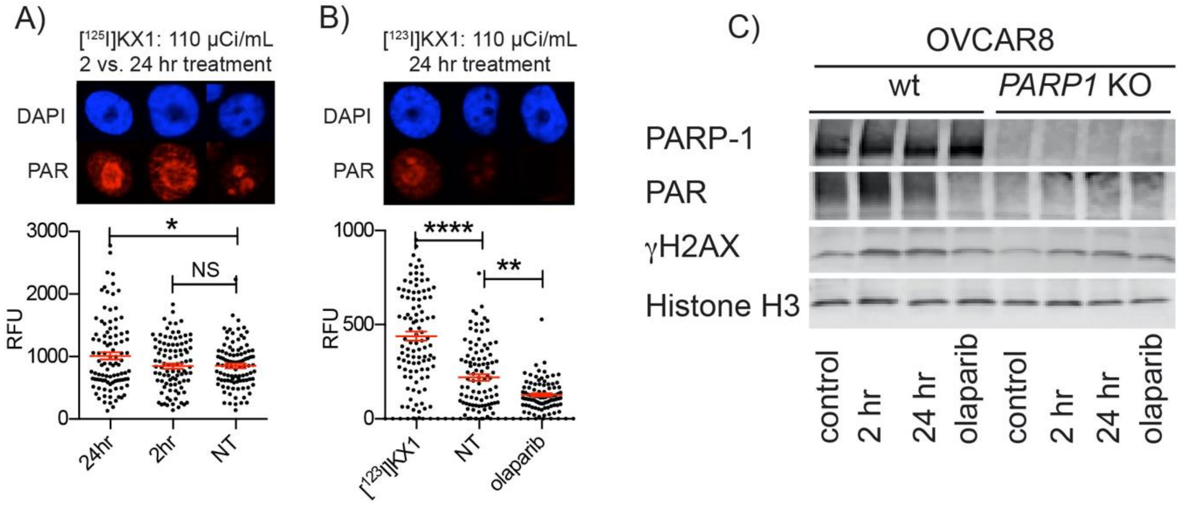

Sup Figure 3. Radiotracer properties were verified by immunofluorescence to demonstrate concentrations required to induce DNA damage were below concentrations for enzymatic inhibition as measured by Poly-ADP-ribose (PAR). **A)** OVCAR8 wildtype cells treated with [^{125}I]KX1 for 2 or 24 h showed no reduction in PAR, although the 24 h time point showed a slight increase. **B)** Similarly, OVCAR8 wildtype cells treated with [^{123}I]KX1 for 24 h showed a significant increase in PAR from non-treated controls and accordingly olaparib treated cells showed a significant decrease. Together these data represent evidence that [$^{123/125}$I]KX1 induce DNA damage at concentrations below PARP-1 enzymatic inhibition. **C)** Western blot analysis of DNA damage and PARP-1 activity in isogenic OVCAR8 wildtype (wt) and *PARP1* knockout (KO) cells treated with [^{125}I]KX1. We found wt cells treated for 2 or 24 hrs did not inhibit PAR (PARP-1 enzymatic activity) but caused an increase in γH2AX (DNA double strand breaks). A clinical PARP inhibitor, olaparib, was used as a positive control and wt cells treated for 24 hrs showed decreased PAR and increased γH2AX. A noticeably lesser effect in DNA damage was observed in *PARP1* KO cells treated with [^{125}I]KX1.

Supplemental Tables

OVCAR8	[^{125}I]KX1	[^{125}I]MIBG	[^{125}I]MIBG + Unlabeled KX1
**Wt**	13.616	496.91	506.16
**Cas9**	12.025	458.43	455.84
**PARP1 KO g1**	46.583	368.409	381.84
**PARP1 KO g2**	36.001	416.62	411.44
**PARP1 KO g3**	57.831	418.47	408.48

Sup Table 1: Effective dose for 50% reduction in survival. Units in kBq/mL. Mean.

	K_D	Bmax	Velaparib K_i	Nuclear Dose (Gy)
**Wt**	7.705	1.277×10^6	9.416×10^{-9}	1.5
**PARP1 KO g2**	7.3	3.9×10^5	1×10^{-8}	1.2

Sup Table 2: Experimental pharmacology values for [^{125}I]KX1 used for estimating nucleus dose.

	[^{125}I]KX1 (Gy)	[^{131}I]KX1 (Gy)
**OVCAR8**	1.41 ± 0.04	8.19 ± 0.11
**OVCAR8 G2**	1.26 ± 0.07	8.77 ± 0.28
**SKOV3**	3.59 ± 0.32	18.7 ± 1.1
**SNU251**	1.68 ± 0.10	4.84 ± 0.23
**UWB1.289**	2.31 ± 0.21	1.62 ± 0.08
**UWB1.289+BRCA1**	3.45 ± 0.36	6.15 ± 0.82
**Mean**	2.3 ± 0.4	8.0 ± 2.4

Sup Table 3: Effective dose for 50% reduction in survival (D_{50}). Mean ± SEM.
